# Estimated Prevalence of Cronkhite-Canada Syndrome, Chronic Enteropathy Associated With *SLCO2A1* Gene, and Intestinal Behçet’s Disease in Japan in 2017: A Nationwide Survey

**DOI:** 10.2188/jea.JE20190349

**Published:** 2021-02-05

**Authors:** Mari S. Oba, Yoshitaka Murakami, Yuji Nishiwaki, Keiko Asakura, Satoko Ohfuji, Wakaba Fukushima, Yosikazu Nakamura, Yasuo Suzuki

**Affiliations:** 1 Department of Medical Statistics, Toho University, Tokyo, Japan; 2 Department of Environmental and Occupational Health, Toho University, Tokyo, Japan; 3 Department of Public Health, Osaka City University Graduate School of Medicine, Osaka, Japan; 4 Department of Public Health, Jichi Medical University, Tochigi, Japan; 5 Department of Internal Medicine, Toho University Sakura Medical Center, Chiba, Japan

**Keywords:** Cronkhite-Canada syndrome, chronic enteropathy associated with *SLCO2A1* gene, intestinal Behçet’s disease, nationwide survey, prevalence

## Abstract

**Background:**

Cronkhite-Canada syndrome (CCS), chronic enteropathy associated with *SLCO2A1* gene (CEAS), and intestinal Behçet’s disease (BD) are classified as intractable intestinal disorders in Japan. However, the national prevalence of these diseases remains unknown. We performed a nationwide survey to estimate the patient numbers and prevalence rates of these diseases throughout Japan in 2017.

**Methods:**

We conducted a mail-based survey targeting hospitals across Japan to estimate the annual numbers of patients with CCS, CEAS, and intestinal BD in 2017. Using a stratified random sampling method, we selected 2,979 hospital departments and asked them to report the number of patients who met specific diagnostic criteria. The total number of patients for each disease was estimated by multiplying the reported numbers by the reciprocal of the sampling rate and response rate. The corresponding prevalence rates per 1,000,000 population were calculated based on the mid-year population of Japan in 2017.

**Results:**

The overall survey response rate was 68.1% (2,029 departments). The estimated numbers of patients with CCS, CEAS, and intestinal BD were 473 (95% confidence interval [CI], 357–589), 388 (95% CI, 289–486), and 3,139 (95% CI, 2,749–3,529), respectively; the prevalence rates per 1,000,000 population were 3.7 (male: 4.0; female: 3.5), 3.1 (male: 3.0; female: 3.1), and 24.8 (male: 24.5; female: 25.0), respectively. The male-to-female ratios were 1.10, 0.94, and 0.93 for patients with CCS, CEAS, and intestinal BD, respectively.

**Conclusions:**

Estimates of the national prevalence of CCS, CEAS, and intestinal BD in Japan were generated and found to be higher than those previously reported.

## INTRODUCTION

Cronkhite-Canada syndrome (CCS), chronic enteropathy associated with *SLCO2A1* gene (CEAS), and intestinal Behçet’s disease (BD) are rare intestinal disorders classified as intractable diseases in Japan. The *Act on Medical Care for Patients with Intractable/Rare Diseases* of 2014 recommends quantifying the national prevalence of such diseases. Disease prevalence is a fundamental epidemiologic indicator that evaluates disease burden in a population and provides health policymakers with important information that can guide efficient resource allocation. Although the prevalence and total numbers of patients with ulcerative colitis and Crohn’s disease (which are also classified as intractable diseases) in Japan have been reported in a nationwide survey,^1^ CCS, CEAS, and intestinal BD have different diagnostic criteria and must be evaluated separately from these diseases.

CCS is characterized by gastrointestinal polyposis, chronic diarrhea, skin hyperpigmentation, hair loss, and nail atrophy.^2^^–^^4^ Numerous case series have been reported in Asian countries.^5^^–^^9^ In Japan, Watanabe et al reported that 213 CCS patients were diagnosed between 2000 and 2013 in 140 teaching hospitals.^10^

CEAS was first reported in 1968^11^ and initially described as chronic nonspecific multiple ulcers of the small intestine.^12^ Its main characteristics are gastrointestinal bleeding and intestinal protein loss due to persistent small intestinal ulcers, but its etiology is unknown and no standard treatment has been established. Umeno et al recently identified loss-of-function mutations in the *SLCO2A1* (ie, Solute Carrier Organic Anion Transporter Family Member 2A1) gene as the possible cause of this disorder,^13^ and its name was revised accordingly. Matsumoto et al previously reported 13 patients treated at two university hospitals and their satellite hospitals between 1964 and 2009,^14^ and more recently detected 63 patients in 2013 through a survey of 58 specialized hospitals.^15^ The same research group then described the clinical features of 46 CEAS patients and highlighted the differences with those of Crohn’s disease.^16^

BD is characterized by episodic inflammation in multiple organs.^17^ Intestinal BD is an uncommon manifestation of this condition, and is prevalent in 12% of Japanese BD cases.^18^ These patients experience refractory gastrointestinal symptoms, such as abdominal pain, diarrhea, gastrointestinal bleeding, and gastrointestinal perforation.^17^ In Japan, 15,284 BD patients were included in a national registry in 2017,^19^ but the number of intestinal BD cases is unclear.

Estimating the prevalence of these rare intestinal disorders would inform and guide public health strategies. To this end, we conducted a nationwide sample survey to estimate the number of patients with CCS, CEAS, and intestinal BD, as well as to measure their prevalence rates per 1,000,000 population in Japan in 2017.

## METHODS

We conducted a nationwide epidemiological survey targeting hospitals (medical institutions with ≥20 beds) to estimate the number of patients with CCS, CEAS, and intestinal BD in the Japanese population. The questionnaire-based survey was performed in 2017 in accordance with the Nationwide Epidemiologic Survey Manual issued by the Research Committee on Epidemiology of Intractable Disease in Japan. The method was identical to that of a previous survey.^1^ Briefly, a stratified random sampling method was used to select departments from all hospitals in Japan. The strata were (1) the type of medical department and (2) the number of beds or hospital type. For the type of medical department, the hospitals were stratified into the following four departments: internal medicine, surgery, pediatrics, and pediatric surgery (hospitals could be counted multiple times). For the number of beds or hospital type, the hospitals were stratified into the following eight categories (with their respective sampling fractions): specialized hospitals (100%), university hospitals (100%), ≥500 beds (100%), 400–499 beds (80%), 300–399 beds (40%), 200–299 beds (20%), 100–199 beds (10%), and ≤99 beds (5%). Specialized hospitals were included as many patients with the target diseases would seek care at these facilities due to the presence of gastrointestinal specialists.

The questionnaire was sent to each sampled department, where physicians were requested to answer three questions regarding the presence of patients with the target diseases, the number of patients with the target diseases (if present), and the number of male patients with the target diseases in 2017. To ensure the accuracy and standardization of patient diagnoses, we included disease-specific diagnostic criteria for CCS, CEAS, and intestinal BD^15^^,^^20^^,^^21^ in the questionnaire envelope. The questionnaire was sent in December 2017, and was re-sent in February 2018 to departments that had not yet responded; a reminder notice was also sent between March and May 2018.

The numbers of patients in 2017 were estimated by multiplying the reported patient numbers by the reciprocal of the sampling rate and survey response rate.^22^ The overall and sex-specific prevalence rates in the Japanese population were estimated using national census statistics in 2017.^23^ Specifically, the mid-year population was used to calculate the prevalence rates per 1,000,000 population.

A sensitivity analysis was performed to assess the potential impact of non-response bias. Specifically, we estimated the minimum number of patients under the assumption that non-responding departments had no patients with CCS, CEAS, or intestinal BD. All statistical sampling and analyses were performed using SAS version 9.4 (SAS Institute, Cary, NC, USA).^24^

The study protocol was reviewed and approved (Approval Number: A17076) by the Ethics Committee of the Faculty of Medicine, Toho University (Tokyo, Japan).

## RESULTS

Table 1 shows the numbers of sampled and responding departments according to medical department type. From a total of 12,326 departments in 8,445 hospitals, 2,979 departments in 1,586 hospitals were sampled through stratified random sampling (internal medicine: 1,050; surgery: 946; pediatrics: 766; and pediatric surgery: 217). The overall response rate was 68.1% (2,029/2,979 departments). Among the bed number or hospital type categories, the response rate was highest in specialized hospitals (88.2%).

**Table 1.** Numbers of sampled and responding departments in the nationwide survey

Department type	Number of beds or hospital type	All departments	Sampled departments	%	Responding departments	%
Internal Medicine	Specialized hospitals	82	82	100%	74	90.2%
	University hospitals	113	113	100%	84	74.3%
	≥500 beds	192	192	100%	106	55.2%
	400–499 beds	223	179	80%	102	57.0%
	300–399 beds	351	141	40%	84	59.6%
	200–299 beds	420	84	20%	41	48.8%
	100–199 beds	1,162	117	10%	68	58.1%
	≤99 beds	2,839	142	5%	83	58.5%

	Subtotal	5,382	1,050		642	61.1%

Surgery	Specialized hospitals	80	80	100%	67	83.8%
	University hospitals	96	96	100%	79	82.3%
	≥500 beds	190	190	100%	126	66.3%
	400–499 beds	213	171	80%	107	62.6%
	300–399 beds	340	136	40%	87	64.0%
	200–299 beds	386	78	20%	44	56.4%
	100–199 beds	987	99	10%	55	55.6%
	≤99 beds	1,907	96	5%	46	47.9%

	Subtotal	4,199	946		611	64.6%

Pediatrics	Specialized hospitals	72	72	100%	63	87.5%
	University hospitals	84	84	100%	78	92.9%
	≥500 beds	178	178	100%	145	81.5%
	400–499 beds	198	159	80%	124	78.0%
	300–399 beds	301	121	40%	93	76.9%
	200–299 beds	304	61	20%	48	78.7%
	100–199 beds	506	51	10%	34	66.7%
	≤99 beds	785	40	5%	19	47.5%

	Subtotal	2,428	766		604	78.9%

Pediatric Surgery	Specialized hospitals	46	46	100%	43	93.5%
	University hospitals	40	40	100%	37	92.5%
	≥500 beds	75	75	100%	57	76.0%
	400–499 beds	39	32	82%	18	56.3%
	300–399 beds	31	13	42%	11	84.6%
	200–299 beds	26	6	23%	3	50.0%
	100–199 beds	22	3	14%	3	100%
	≤99 beds	38	2	5%	0	0.0%

	Subtotal	317	217		172	79.3%

Total	Specialized hospitals	280	280	100%	247	88.2%
	University hospitals	333	333	100%	278	83.5%
	≥500 beds	635	635	100%	434	68.3%
	400–499 beds	673	541	80%	351	64.9%
	300–399 beds	1,023	411	40%	275	66.9%
	200–299 beds	1,136	229	20%	136	59.4%
	100–199 beds	2,677	270	10%	160	59.3%
	≤99 beds	5,569	280	5%	148	52.9%

Overall		12,326	2,979		2,029	68.1%

Table 2 presents the estimated numbers of patients with CCS in 2017 according to medical department type. There were 473 patients (95% confidence interval [CI], 357–589), with 248 (95% CI, 210–285) male patients and 225 (95% CI, 117–334) female patients. There were no patients with the target diseases in the pediatric or pediatric surgery departments. Table 3 presents the estimated numbers of patients with CEAS according to medical department type. There were 388 patients (95% CI, 289–486), with 188 (95% CI, 128–248) male patients and 200 (95% CI, 128–272) female patients. Five pediatric departments and two pediatric surgery departments responded that they had treated patients with this disease. The proportion of CEAS patients reported from pediatric or pediatric surgery departments was 10.3%. Table 4 presents the estimated numbers of patients with intestinal BD according to medical department type. There were 3,139 patients (95% CI, 2,749–3,529), with 1,514 (95% CI, 1,293–1,735) male patients and 1,625 (95% CI, 1,366–1,885) female patients. The proportion of intestinal BD patients reported from pediatric or pediatric surgery departments was 3.0%.

**Table 2.** Estimated numbers of patients with Cronkhite–Canada syndrome in Japan in 2017

Department type	Overall		Male		Female	
	Number of patients	95% confidence interval	Number of patients	95% confidence interval	Number of patients	95% confidence interval
Internal medicine	384	301–466	222	186–258	162	88–235
Surgery	89	8–170	25	15–36	64	0–144
Pediatrics	0					
Pediatric surgery	0					

Total	473	357–589	248	210–285	225	117–334

**Table 3.** Estimated numbers of patients with chronic enteropathy associated with *SLCO2A1* gene in Japan in 2017

Department type	Overall		Male		Female	
	Number of patients	95% confidence interval	Number of patients	95% confidence interval	Number of patients	95% confidence interval
Internal medicine	289	203–375	133	46–219	156	87–225
Surgery	58	34–84	32	10–54	26	16–38
Pediatrics	36	0–77	20	0–45	16	0–32
Pediatric surgery	4	0–8	3	0–7	1	1–2

Total	388	289–486	188	128–248	200	128–272

**Table 4.** Estimated numbers of patients with intestinal Behçet’s disease in Japan in 2017

Department type	Overall		Male		Female	
	Number of patients	95% confidence interval	Number of patients	95% confidence interval	Number of patients	95% confidence interval
Internal medicine	2,385	2,067–2,703	1,141	1,002–1,279	1,244	1,021–1,467
Surgery	661	436–885	330	159–501	331	198–463
Pediatrics	87	65–110	39	26–53	48	30–66
Pediatric surgery	7	3–10	4	1–7	3	2–4

Total	3,139	2,749–3,529	1,514	1,293–1,735	1,625	1,366–1,885

The prevalence rates of CCS, CEAS, and intestinal BD per 1,000,000 population based on the mid-year population of Japan in 2017 were 3.7 (male: 4.0 and female: 3.5), 3.1 (male: 3.0 in men and female: 3.1), and 24.8 (male: 24.5 and female: 25.0), respectively. The male-to-female ratios were 1.10 for CCS, 0.94 for CEAS, and 0.93 for intestinal BD. There were no substantial differences in sex distribution for any of the target diseases.

In the sensitivity analysis, non-responding departments were assumed to have no patients with the target diseases. Under this assumption, the estimated numbers of patients were approximately 0.7 times that of the base-case analysis (eTable 1).

## DISCUSSION

This study estimated the numbers of patients with rare intestinal disorders in Japan in 2017 using a nationwide sample survey. Our study estimated that there were 473 patients with CCS, 388 patients with CEAS, and 3,139 patients with intestinal BD throughout Japan.

Our estimated numbers of patients with the target diseases were higher than those of previous reports. For example, Watanabe et al reported that there were 213 CCS patients treated in Japanese teaching hospitals,^10^ which was less than half of our estimate. A research group for rare and intractable intestinal disorders performed a survey in 2013, and estimated that there were approximately 160 CEAS patients based on 63 patients identified from 58 specialized hospitals.^15^ The discrepancy between our results and those of previous studies may be due to the difference in target populations. Previous studies on CCS and CEAS mainly focused on larger hospitals or teaching hospitals and did not include all types of hospitals. To further explore this discrepancy, we restricted the hospital types in our analysis to those used in previous studies (ie, specialized hospitals, university hospitals, and large hospitals with ≥500 beds) and estimated the number of patients. This emulation resulted in estimates of 266 and 200 patients with CCS and CEAS, respectively, which were closer to those of previous studies. This indicates that our approach of analyzing all hospital types—including smaller and non-specialized hospitals—provided a more representative estimate of these patients in Japan. On the other hand, our approach may also have incorporated disease misclassifications. Since previous reports counted the number of patients diagnosed by specialists in specialized hospitals, their diagnoses are likely to be clinically accurate. As we conducted a mail-based survey in over 2,000 hospital departments throughout Japan, we could not confirm the accuracy of each diagnosis. Nevertheless, we had enclosed specific diagnostic criteria with the questionnaires to minimize such misclassifications.

The numbers of patients enrolled in the national registry for intractable diseases in Japan were 108, 65, and 15,284 patients with CCS, CEAS, and BD, respectively in 2017. Intestinal BD was estimated to be 10% of them.^18^ Those numbers were also smaller than our estimates. Registration in such registries is incentivized by the subsidization of treatment costs, and there may be patients who do not register because they do not need financial support. In addition, patients with milder forms of the disease may have a lower registration rate. The number of patients enrolled in this registry has increased yearly since 2015 following the enactment of the *Act on Medical Care for Patients with Intractable/Rare Diseases*,^19^ and this increase may continue in the future.

Formal surveys of CCS have yet to be reported in other countries. In Asia, She et al collected and described case reports of 50 CCS patients in China,^9^ and Yun et al reported on 13 CCS cases in South Korea.^8^ In the United States, the Mayo Clinic reported detailed information from 14 CCS patients treated from 1955 to 2009.^25^ Our search of the literature found no other studies that reported the number of CCS patients, which reinforces the notion that this is an extremely rare disease globally. CEAS is considered to be identical to cryptogenic multifocal ulcerous stenosing enteritis in western countries, but the prevalence of the latter has rarely been reported. The exception is Perlemuter et al, which identified 12 patients hospitalized between 1965 and 1993 in France.^26^ However, their survey-based study had a response rate of only 3%. BD is thought to be more prevalent along the Silk Road, with a high prevalence in Turkey and low prevalences in Europe and North America.^17^ Recent diagnostic criteria for BD do not require gastrointestinal involvement,^27^ and the global prevalence of intestinal BD is, therefore, unclear. However, Kirino et al^18^ and Kim et al^28^ reported that the proportion of intestinal BD is approximately 10% in Japan and South Korea, respectively, and is continuing to increase.

The estimated male-to-female ratios in this study were closer to one (ie, more evenly distributed) than previously reported. Studies have estimated the male-to-female ratio to be 1.8 (136 male and 74 female patients)^10^ and 1.3 (31 male and 24 female patients)^3^ among CCS patients, and 0.4 (4 male and 11 female patients)^12^ and 0.4 (13 male and 33 female patients)^16^ among CEAS patients. Nevertheless, the directions of the sex distributions were similar between these previous studies and our findings. We believe that our probabilistic approach involving a large number of hospitals provides more accurate estimates than previous surveys.

The strength of our study lies in its use of a standardized protocol developed collaboratively by epidemiologists and biostatisticians.^22^ As our study targeted all Japanese hospitals, our estimate can be interpreted to represent the total number of patients in Japan. The accuracy of a survey’s estimates is dependent on its response rate, and ours was relatively high (68.1%) due in part to the re-sending of questionnaires and follow-up queries. Furthermore, we also performed a sensitivity analysis that assumed an extreme scenario (in which non-responding department had no relevant patients) to confirm the minimum number of patients with the target diseases. The sensitivity analysis indicated that in such a scenario, the numbers of patients were approximately 0.7 times that of the base-case analysis.

This study has several limitations. First, our analysis did not consider possible duplicate counts of patients who sought care at multiple departments. In a survey of ulcerative colitis and Crohn’s disease conducted in 1991, duplicate patients comprised only 0.9% of the sample and their influence appeared to be small.^29^ Second, we did not include clinics (defined in Japan to be medical institutions with ≤19 beds) in our survey. This may have led to an underestimation of patient numbers. However, patients with these rare intestinal disorders in Japan are generally referred to larger hospitals to confirm their diagnoses and receive treatment. Therefore, we believe that the non-inclusion of clinics would have a negligible effect on our findings. Third, our analysis did not estimate the incidence of these diseases. Although incidence is important from both the clinical and public health perspectives, there are difficulties in estimating the incidences of rare diseases using a probabilistic approach. Fourth, we did not mention the issue of the designated medical institutions for intractable diseases in this study. In our study, departments in large hospitals, university hospitals, and specialized hospitals were all included (100%) in sampled departments. These departments mostly belonged to designate medical institution and we were sure that we did not miss the designated medical institutions in these categories. Meanwhile, it is not certain whether a small hospital in our study was a designated medical institution or not. However, as we selected medical institutions randomly in small hospitals strata, the proportion of designated medical institutions in our sample was equal to that in all medical institutions in Japan. So, the estimated numbers of patients were not biased by the designation of medical institutions under the assumption that the response was random. Fifth, we did not collect age information of the patients in our survey. We only know the proportion of patients reported from pediatric or pediatric surgery departments and those were low especially among CCS and intestinal BD.

Despite these limitations, our study provides accurate estimates of the patient numbers and prevalence rates of CCS, CEAS, and intestinal BD. These are important indicators of disease burden, and may help in the planning of future research and development of care.

### Conclusions

Based on a nationwide survey of rare intestinal disorders using a standardized protocol, our study estimated that there were 473 CCS patients, 388 CEAS patients, and 3,139 intestinal BD patients in Japan in 2017. These estimates are substantially higher than those of previous reports and provide a more representative overview of the disease burden for these rare conditions.

## APPENDIX A. SUPPLEMENTARY DATA

eTable 1. Sensitivity analysis: estimated numbers of patients with CCS, CEAS, and intestinal BD in Japan in 2017 under the assumption that non-responding departments had no corresponding patients
