# Supplementary material for: Estimated Prevalence of Cronkhite-Canada Syndrome, Chronic Enteropathy Associated With SLCO2A1 Gene, and Intestinal Behçet’s Disease in Japan in 2017: A Nationwide Survey
Source: J Epidemiol. 2021 Feb 5;31(2):139–44. doi: 10.2188/jea.JE20190349 (PMC7813772; doi:10.2188/jea.JE20190349)
Supplement: Supplementary file 1 [file je-31-139-s001.pdf]

eTable 1. Sensitivity analysis: estimated numbers of patients with CCS, CEAS, and intestinal BD in Japan in 2017 under the assumption that non-responding departments had no corresponding patients

Disease	Overall number of patients	95% confidence interval
CCS	309	251–366
CEAS	277	218–355
Intestinal BD	2,200	2,003–2,398

BD, Behçet’s disease; CCS, Cronkhite–Canada syndrome; CEAS, chronic enteropathy associated with *SLCO2A1* gene.
